# Understanding Degradation at the Lithium-Ion Battery Cathode/Electrolyte Interface: Connecting Transition-Metal Dissolution Mechanisms to Electrolyte Composition

**DOI:** 10.1021/acsami.0c22235

**Published:** 2021-03-04

**Authors:** Di Huang, Chaiwat Engtrakul, Sanjini Nanayakkara, David W. Mulder, Sang-Don Han, Meng Zhou, Hongmei Luo, Robert C. Tenent

**Affiliations:** † National Renewable Energy Laboratory, Golden, Colorado 80401, United States; ‡ Department of Chemical and Materials Engineering, New Mexico State University, Las Cruces, New Mexico 88003, United States; § Renewable and Sustainable Energy Institute, University of Colorado, Boulder, Colorado 80303, United States

**Keywords:** scanning electrochemical microscopy, cathode/electrolyte interface, LiMn_2_O_4_, polymer-assisted deposition, Mn dissolution

## Abstract

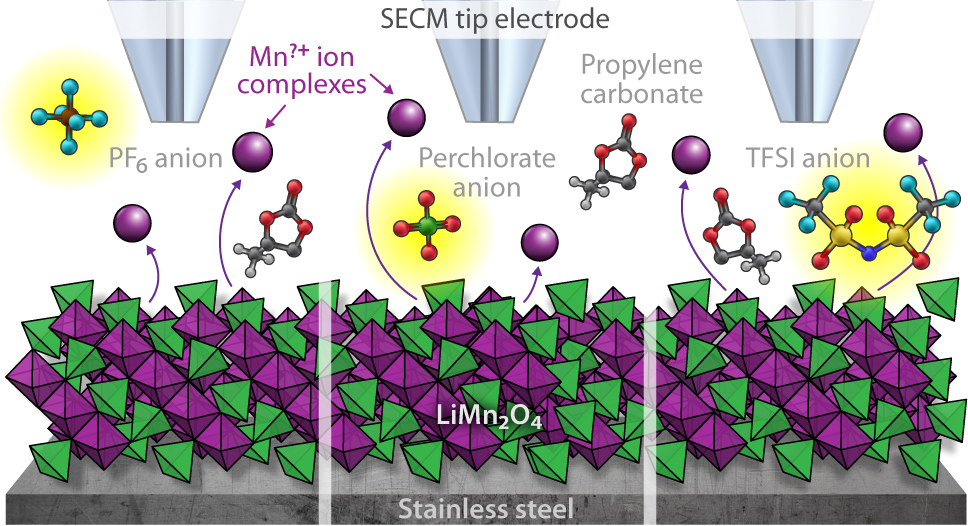

Lithium transition-metal oxides (LiMn_2_O_4_ and LiMO_2_ where M = Ni, Mn, Co, *etc.*) are widely applied as cathode materials in lithium-ion batteries due to their considerable capacity and energy density. However, multiple processes occurring at the cathode/electrolyte interface lead to overall performance degradation. One key failure mechanism is the dissolution of transition metals from the cathode. This work presents results combining scanning electrochemical microscopy with inductively coupled plasma (ICP) and electron paramagnetic resonance (EPR) spectroscopies to examine cathode degradation products. Our effort employs a LiMn_2_O_4_ (LMO) thin film as a model cathode to monitor the Mn dissolution process without the potential complications of conductive additive and polymer binders. We characterize the electrochemical behavior of LMO degradation products in various electrolytes, paired with ICP and EPR, to better understand the properties of Mn complexes formed following metal dissolution. We find that the identity of the lithium salt anions in our electrolyte systems [ClO_4_^–^, PF_6_^–^, and (CF_3_SO_2_)_2_N^–^] appears to affect the Mn dissolution process significantly as well as the electrochemical behavior of the generated Mn complexes. This implies that the mechanism for Mn dissolution is at least partially dependent on the lithium salt anion.

## 1 Introduction

Due to the combination of light weight and high power density, lithium-ion batteries (LIBs) have become the power source of choice for a wide variety of applications and the leading technology driving the electrification of vehicles.^1−4^ While highly successful in consumer electronics such as laptop computers and cell phones, the performance of LIBs still lags behind requirements for broad adoption in the electric vehicle (EV) market. As an example, the useable lifetime of present LIB technologies falls well short of the projected goal for broad EV adoption of 15 years, as defined by the United States Advanced Battery Consortium.^5^ Multiple processes occurring at the cathode/electrolyte interface, such as metal dissolution and oxygen evolution, are known to lead to long-term cell life issues for LIBs.^6,7^ However, key mechanistic details of how these processes occur remain unclear and must be better understood to improve long-term performance.

The present model for Mn dissolution from LiMn_2_O_4_ (LMO), as shown in eq 1, was proposed by Gummow *et al.* based on the previous work of Hunter, who examined the chemical delithiation of LMO in acidic, aqueous solutions.^8,9^
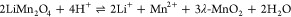
1

This reaction has historically been used as a model for the Mn dissolution process, especially in the presence of HF (a side product of hydrolysis of the commonly used PF_6_^–^ anion). However, as multiple authors have pointed out, it is not intuitively clear that the proposed aqueous phase mechanism is applicable in a nonaqueous system. This has led to significant research on the mechanism of Mn dissolution in LIB electrolytes with multiple competing mechanisms now proposed.^10,11^ Recently, Wang *et al.* demonstrated that the Mn^2+^ ion believed to be generated in the dissolution process can easily react with various components of the electrolyte, including commonly used carbonate solvent molecules and electrolyte anions.^12^ This process likely leads to additional reactions of Mn species following evolution from the cathode surface. The Mn complexes resulting from the dissolution process and potential subsequent solution-phase reactions are then deposited onto the solid–electrolyte interface of the anode driving cell degradation, also by still debated mechanisms.^13^ To gain further insights into how these Mn complexes drive cell degradation, it is important to study their properties and how they may change at varying points as they evolve from the cathode, potentially react with the electrolyte, and ultimately deposit at the anode surface.

In this paper, we report a multipronged effort employing a combination of *in situ* and *ex situ* methods to study the properties and reactivity of Mn complexes emerging from a model LMO cathode material (without organic binders and conductive carbon additives) in an active electrochemical cell. These methods included electron paramagnetic resonance (EPR) and inductively coupled plasma (ICP) spectroscopies as well as electrochemical methods, including scanning electrochemical microscopy (SECM). ICP enabled quantitation of Mn in LIB electrolytes, while EPR was used as a direct measurement of the formation of Mn ions in the electrolytes, using a method previously described by Banerjee *et al.*(14) SECM was used to study the electrochemical reactivity of LMO degradation products both near the cathode/electrolyte interface as well as in bulk electrolyte solution.

## 2 Experimental Section

### 2.1 Chemicals

Lithium nitride (LiNO_3_), manganese (II) acetate tetrahydrate (Mn [CH_3_CO_2_]_2_·4H_2_O), polyethylenimine (PEI), ethylenediaminetetraacetic acid (EDTA), and lithium bis(trifluoromethanesulfonyl)imide (LiTFSI) were purchased from Sigma-Aldrich. Manganese bis(trifluoromethanesulfonyl)imide (#47179, Mn(TFSI)_2_) was purchased from Alfa Aesar. 1 M lithium perchlorate (LiClO_4_) in propylene carbonate (PC) was purchased from Novolyte Technologies, part of the BASF Group. 1 M lithium hexafluorophosphate (LiPF_6_) in PC was purchased from Sigma-Aldrich. 1 M LiTFSI in PC was prepared by dissolving LiTFSI in pure PC (Sigma-Aldrich). The water content of all electrolytes was measured using the Karl Fischer titration method. Water content in our LiClO_4_, LiPF_6_, and LiTFSI-based electrolytes were found to be 5.5 ppm, 8.3 ppm, and 7.1 ppm, respectively.

### 2.2 Preparation of LiMn_2_O_4_ Substrates

LMO films were prepared by the polymer-assisted deposition (PAD) method. This method has been previously applied to fabricate various metal oxides, including Li(Ni_*x*_Co_*y*_Mn_*z*_)O_2_.^15−18^ For LMO films, lithium nitride and manganese acetate tetrahydrate were dissolved in deionized water at 1.1:1 (Li/Mn molar ratio) to prepare the metal salt solution. A 10% excess lithium was added to compensate for the evaporation of lithium during calcination. EDTA and PEI (50 wt %) were mixed at a 1:2 mass ratio and dissolved in deionized water to prepare the polymer solution. The polymer solution and metal salts solution were mixed together and stirred until the solution was homogeneous to obtain the final LMO precursor solution.

Stainless steel (SS) 304 substrates were purchased from MTI. The SS substrate was cleaned in acetone, sonicated for 20 min, and then rinsed with acetone and deionized water. After drying in air, the SS substrate was cleaned in an ozone cleaner for 15 min. Approximately 30 μL of the LMO precursor solution was spin coated onto the substrate at 3000 rpm for 30 s. Eventually, the films were transferred into a furnace and calcined from room temperature to 600 °C (1 °C/min) and held at 600 °C for 1 h in air.

### 2.3 Physical Characterization

X-ray diffraction (XRD) was carried out using an Empyrean series 2, PANalytical instrument with a Cu Kα radiation of 1.54059 Å at 45 kV and 40 mA within the range of 10–80° (2θ) to determine whether the correct crystal structures (spinel LiMn_2_O_4_) and impurity phases were obtained. Scanning electron microscopy (SEM) was evaluated by Hitachi-S4800 to reveal the surface morphology of thin films and assess the quality of LMO thin films. ICP–MS was employed to determine the Mn concentration in the electrolytes with measurements provided by Huffman Hazen Laboratories, Golden, CO. Samples were analyzed using a PerkinElmer ICP–MS instrument. The manganese signal was monitored at mass to charge (*m*/*z*) of 55 amu. An internal standard of rhodium was added in-line to the sample introduction system to monitor instrument stability throughout the analysis run. Each sample was analyzed two times with relative standard deviation between the measurements of at most ±10%.

### 2.4 Electrochemical Characterization

#### 2.4.1 Half-Coin Cell

The as-prepared LMO film on SS was directly used as a cathode electrode after heating for 15 min to remove moisture from the LMO film’s surface. Coin cells were assembled in an argon gas-filled glovebox (<0.5 ppm of H_2_O and <0.5 ppm of O_2_) with the LMO film working electrode, electrolyte (1 M LiPF_6_ in ethylene carbonate (EC):dimethyl carbonate (DMC), 1:1 in volume ratio), separator (Celgard 2300), and a lithium metal counter/reference electrode. The electrochemical behavior was evaluated by using BTS4000 from Neware Technology after a 6 h rest.

#### 2.4.2 SECM

The SECM setup was housed in an argon-filled gloved box (<0.5 ppm of H_2_O and <0.5 ppm of O_2_). LMO films prepared by PAD were used as the substrate electrodes. A 25 μm diameter Pt wire-embedded disk electrode was used as the SECM tip electrode. A Pt wire was used as a counter electrode, while lithium foil was used as a quasi-reference electrode. Three lithium electrolytes were examined: 1 M LiClO_4_ in PC, 1 M LiPF_6_ in PC, and 1 M LiTFSI in PC. The electrochemical properties were analyzed by an electrochemical workstation integrated with the CHI920C SECM from CH Instruments, Inc.

#### 2.4.3 Generation/Collection SECM

The LMO film was charged at a constant voltage of 4.5 V (*vs* Li/Li^+^, hereafter), accelerating the Mn dissolution into the electrolyte, while the tip was positioned above the substrate to monitor the species generated during charging. In some cases, the Pt tip was positioned ∼20 μm above the substrate, as described in the Supporting Information. In other experiments, the tip was held several millimeters above the LMO substrate to characterize species occurring in bulk solution far from the active LMO surface. A cyclic voltammogram (CV) was collected at the SECM Pt tip electrode at the scan rate of 1 V/s before and after the LMO substrate was held at 4.5 V for a predetermined amount of time. The LMO substrate was kept unbiased during the tip CV measurement.

#### 2.4.4 EPR Spectroscopy

EPR measurements were performed using a Bruker X-band ELEXSYS E-500 CW EPR spectrometer equipped with an in-cavity cryostat (Bruker/ColdEdge Technologies). The magnetic field was calibrated with a 2,2-diphenyl-1-picrylhydrazyl crystalline sample. All measurements were recorded at room temperature using a Bruker super-high-Q resonator (ER4122SHQE/0113). Approximately 30 μL of the sample was loaded into a quartz capillary tube (Wilmad-LabGlass, WG-221T-RB), which was sealed with epoxy and placed inside an X-band EPR tube (Wilmad-LabGlass, 707-SQ-250M). Typical EPR parameters were as follows: microwave power, 1 mW; modulation frequency, 100 kHz; modulation amplitude, 10 G; time constant, 81.92 ms; and conversion time, 81.92 ms. All spectra were manually baseline corrected.

## 3 Results and Discussion

### 3.1 Synthesis and Characterization of Model Cathode Materials

Thin-film LMO was prepared as a model cathode material by PAD. PAD is a simple solution phase process to deposit a wide variety of metal oxide materials^15,19,20^ with pure phase, low roughness, and high uniformity over large areas.

**Figure 1:**
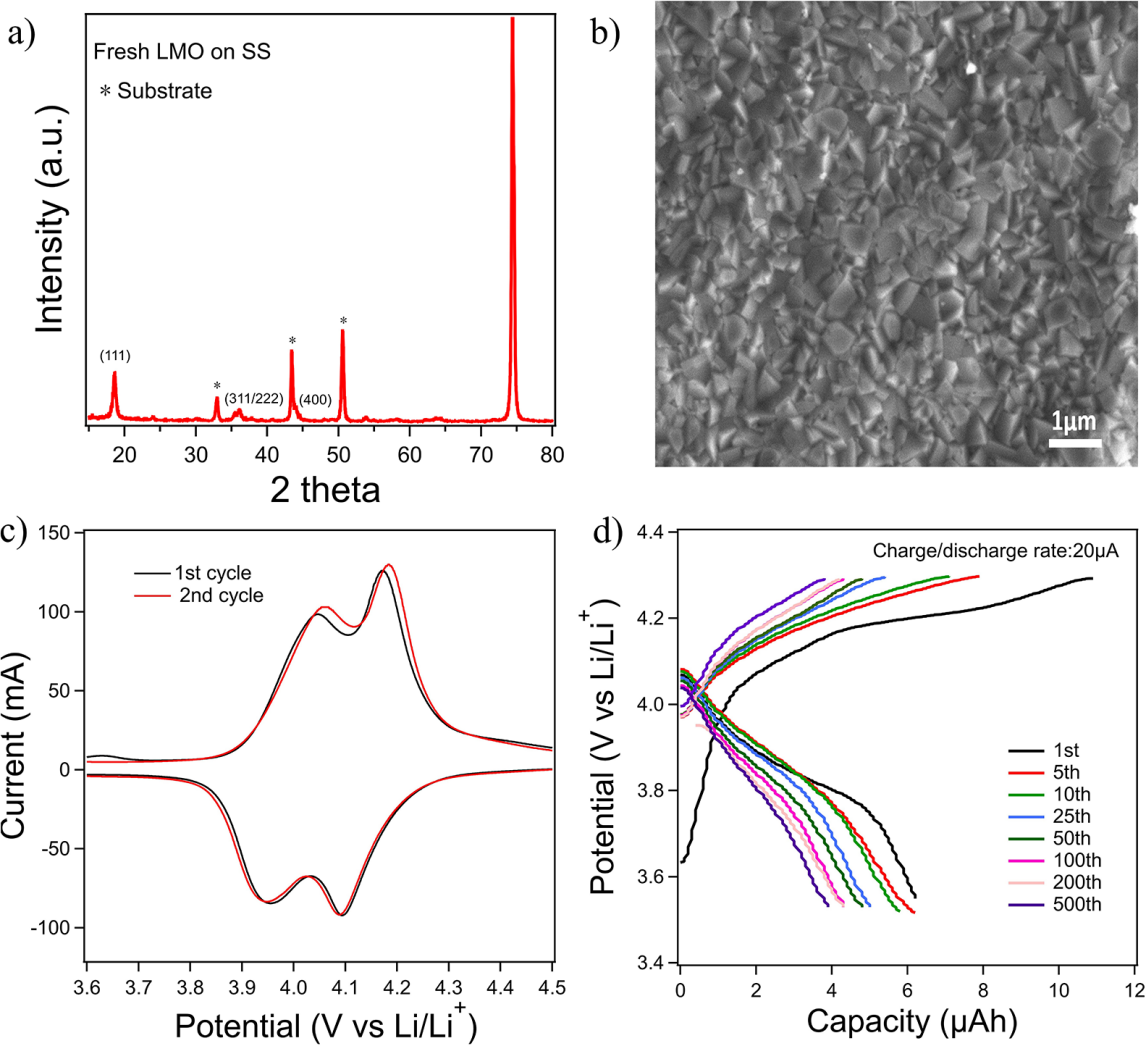
(a) XRD of a fresh LiMn_2_O_4_ film on SS, (b) SEM image of the LiMn_2_O_4_ film surface (top view), (c) cyclic voltammetry of an as-prepared half-cell using LiMn_2_O_4_ as the working electrode (*vs* a Li metal counter/reference electrode) in LiPF_6_ in EC/DMC at 0.1 mV/s, and (d) galvanostatic charge/discharge curves of the as-prepared half-cell.

LMO has a spinel structure with space group of *Fd3̅m*. In the XRD pattern, as shown in Figure 1a, peaks at around 18, 36, and 44° are attributed to the spinel LMO (111), (311/222), and (400), respectively, which are well matched with standard JCPDS card no. 88-1026, indicating the formation of the LMO spinel phase.^21−23^ Additional peaks present are related to the SS substrate. Figure 1b demonstrates a top view SEM image of an LMO film on SS showing a dense film consisting of small crystal particles with sizes of less than 500 nm. To verify functionality of the PAD LMO films, we evaluated the electrochemical behavior in a half-coin cell format. Figure 1c shows cyclic voltammetry data for the LMO half-cell at the scan rate of 0.1 mV/s from 3.4 to 4.6 V versus Li/Li^+^. The double redox peaks observed indicate the expected two-step reversible lithiation and delithiation processes between LMO and λ-MnO_2_,^22,24,25^ demonstrating the functionality of the binder-free LMO films as a cathode. Figure 1d shows the charge/discharge curves for the LMO on the SS substrate up to 500 cycles. Two voltage plateaus in the first cycle at around 4 V agree well with the two pairs of redox peaks in the CV curve. As shown in Figure S1, the initial charge and discharge capacities are around 11–6 μA h with a low initial Coulombic efficiency of 55%. After 20 cycles, the efficiency rapidly increased to nearly 100%. After 500 cycles, the thin film electrode can still deliver a reversible capacity of 5 μA h, proving acceptable cycling stability. Figure S1 also demonstrates the rate performance of our model cathode that was carried out by galvanostatic charging and discharging at 20, 40, 60, 100, and 200 μA and back to 20 μA. The LMO thin film electrode shows excellent rate capability as capacity was fully recovered to its initial value as the rate reversed back to 20 μA. These results show that the LMO films prepared by PAD are acceptable representations of cathode materials and well-suited for studying fundamental Mn dissolution mechanisms.

### 3.2 Evaluation of Cathode Degradation Products by SECM

**Figure 2:**
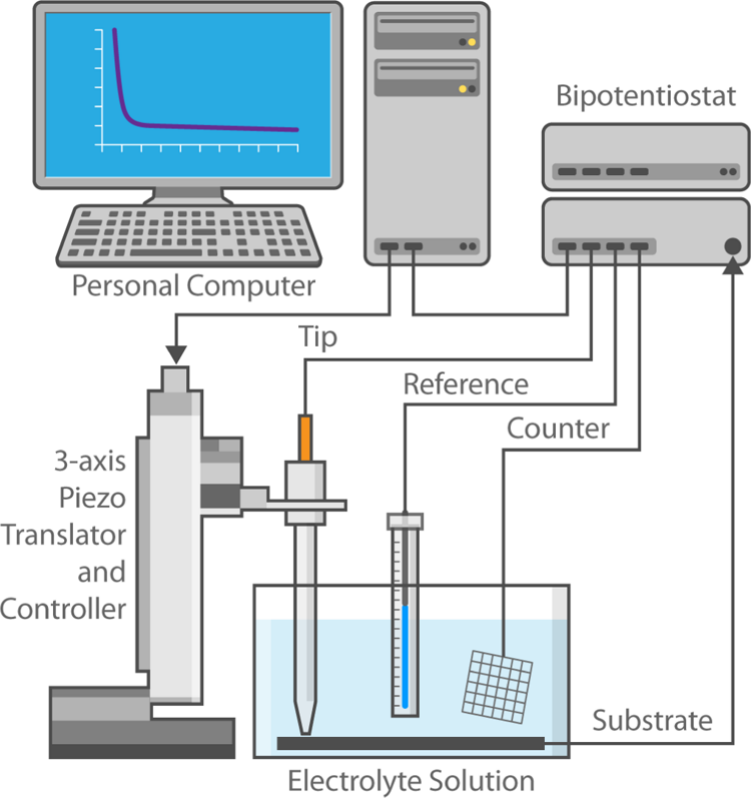
SECM configuration.

*In situ* electrochemical analysis was conducted using SECM, which has been described previously.^26−30^ In brief, SECM, as shown in Figure 2, is scanning probe microscopy that can be used to perform electrochemical analysis of species evolving from or being consumed at an electrode surface. Analysis occurs by using a small electrode as a sensing “tip” in an active four-electrode electrochemical cell containing an electrolyte solution. The applied potential at both the substrate and the tip electrode is controlled versus the same reference and counter electrodes by a bi-potentiostat. The tip electrode commonly consists of a conductive wire (micron—nanometer in diameter) embedded in glass and ground/polished to a point. The instrument can be used both as an imaging technique, as implied by its name, as well as a measurement technique. The work presented here focuses on the application of SECM as an *in situ* measurement system in the so-called generation/collection (G/C) mode. In G/C mode SECM, the tip electrode is used to conduct experimental measurements of species evolving from a substrate sample surface in a manner similar to the rotating ring-disk electrode method. In this work, the tip electrode is used to perform *in situ* electrochemical analysis at varying distances from the underlying LMO substrate. The use of a small tip electrode for characterization enables measurements to be made very near the active cathode/electrolyte interface, allowing the examination of short-lived transient species that may only be observed prior to further solution phase reactions.

Typical SECM analysis was conducted in an electrochemical cell consisting of the model LMO cathode material as a substrate, a 25 μm diameter Pt disk electrode as a tip with a lithium metal quasi-reference electrode, and a platinum counter electrode. Degradation of LMO was accelerated by holding the substrate at 4.5 V versus Li/Li^+^, which is known to drive dissolution of Mn from LMO^31^ for varying lengths of time. *In situ* electrochemical analysis of species evolving from the degrading LMO film was conducted using cyclic voltammetry conducted at the tip before and after exposing the substrate to the high voltage hold.

### 3.3 Characterization of Mn Dissolution from PAD LMO

**Figure 3:**
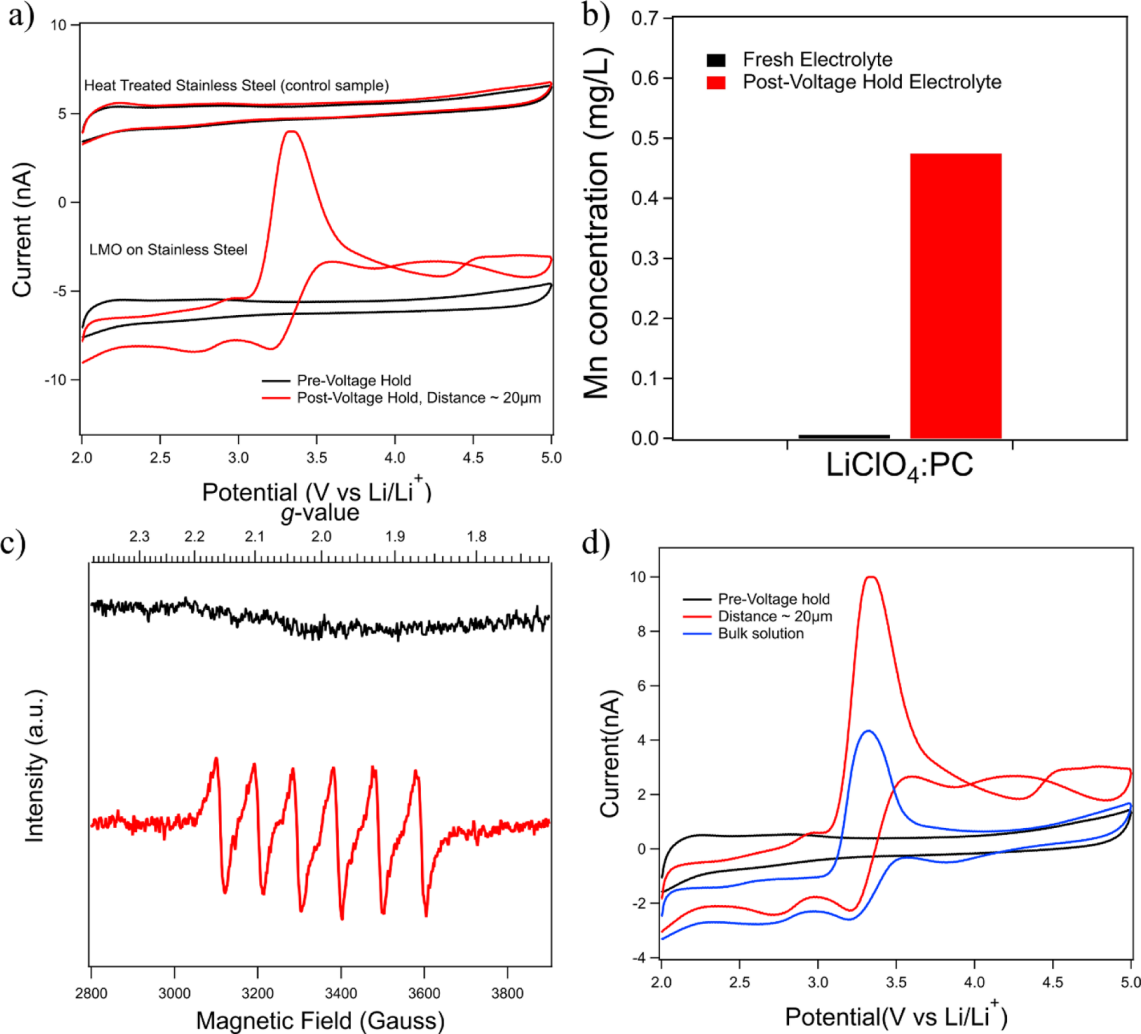
(a) SECM tip electrode voltammograms collected over an LMO substrate (bottom) and heat-treated SS (top) with tip held above the substrate in LiClO_4_/PC at the scan rate of 1 V/s using a 25 μm Pt tip. (b) ICP of fresh and post voltage hold LiClO_4_/PC. (c) EPR signals for LiClO_4_/PC before (black) and after voltage hold (red). (d) SECM tip electrode voltammograms collected over the LMO substrate in bulk solution (blue) and ∼20 μm (red) above the LMO substrate at the scan rate of 1 V/s using a 25 μm Pt tip.

Figure 3 shows a combination of ICP, EPR, and SECM data for the analysis of LMO degradation in 1 M LiClO_4_ dissolved in PC (LiClO_4_/PC). Cyclic voltammetry data were collected at the tip electrode both before and after exposing the LMO substrate to the 4.5 V hold. ICP and EPR studies were conducted on electrolytes harvested from the SECM cell following the high voltage LMO hold as well as the unexposed electrolyte for comparison.

Figure 3a shows G/C SECM voltammograms collected for experiments conducted with the aforementioned LMO substrate as well as an uncoated SS substrate processed identically to the LMO sample. The tip electrode was placed ∼20 μm away from the LMO substrate surface using a SECM negative feedback approach process, as described in the Supporting Information (please see Figure S2). CVs were then collected at the tip electrode before and after the application of a 4.5 V hold on the substrate for a total of 5 h. The top two traces show data collected near the heat-treated SS substrate control sample before (black trace) and after (red trace) the high voltage hold. Note that data for the SS control sample show an identical voltammetric response before and after the high voltage hold. This shows that no electrochemically active species are formed from reactions of the SS substrate with the electrolyte during the high voltage hold. In contrast, the lower portion of Figure 3a shows data from the companion experiment using our model LMO cathode on SS as the substrate. Here, again, the black trace represents the initial background scan of the cell electrolyte, while the red traces show data collected after the 5 h, 4.5 V hold. Note that following the high voltage hold in the presence of the LMO substrate, multiple voltammetric features emerge. A significant oxidation peak is observed at ∼3.3 V with reduction peaks at ∼3.2 and 2.7 V. In addition, a further oxidation process is observed at ∼4.5 V and a potentially associated reduction peak is observed at 3.9 V versus Li/Li^+^. In addition, the return sweep following the oxidation process at 4.5 V actually crosses back over the original scan, potentially indicating an interaction with the underlying substrate surface, although the origin of this effect remains unclear.

Figure 3b,c shows data for ICP and EPR studies of electrolyte samples collected from the SECM cell, following the above-discussed experiment. For comparison, ICP and EPR were also collected for fresh electrolyte samples that were never exposed to the LMO substrates. ICP data show that nearly 0.5 mg/L Mn was found in the electrolyte sample after the voltage hold, demonstrating the clear presence of Mn evolving from the LMO electrode. EPR was used to investigate the oxidation state of Mn ions in the electrolyte sample (^55^Mn, nuclear spin *I* = 5/2). For the different oxidation states, Mn^3+^ (3d^4^, *S* = 2) is EPR silent and tends to exhibit large zero-field splitting, while both Mn^2+^ (3d^5^, *S* = 5/2, *I* = 5/2) and Mn^4+^ (3d^3^, *S* = 3/2, *I* = 5/2) are EPR active at the conventional X-band frequency (9.38 GHz).^32^Figure 3c shows EPR spectra collected for the electrolyte harvested from the SECM cell following a voltage hold experiment and comparison to the fresh electrolyte. The data collected after the high voltage hold show six hyperfine lines with a hyperfine coupling constant (*A*) of 98 G (Gauss) and *g* = 2.008, indicating the presence of Mn^2+^ in the electrolyte post voltage hold. These values are in good agreement with those reported by Bard *et al.* for Mn(ClO_4_)_2_ in dimethylformamide^33^ and more generally for Mn^2+^ ions, which typically display a *g*-value > 2.000.^34,35^ Overall, the EPR signal in Figure 3c did not show signs of Mn^4+^, which is ruled out due to its lower *g*-value (*g* = 1.994)^34,35^ and more narrow hyperfine structure (*A* < 80 G), compared to Mn^2+^ in an octahedral environment.^36,37^ It should be noted that Banerjee *et al.* also concluded that the fraction of Mn^4+^ in all electrolyte samples harvested from lithium manganese graphite cells was zero.^14^ Future studies will include investigating the zero field splitting parameters (*E* and *D*) for Mn^2+^ and Mn^4+^ to further elucidate the Mn-oxidation state in LIB electrolyte samples.^38^ Based on the confirmation of the presence of Mn in the electrolyte following the high voltage hold by both ICP and EPR as well as EPR identification of the oxidation state as Mn^2+^, we have tentatively assigned the initial oxidation process observed at ∼3.3 V to the oxidation of Mn^2+^ to Mn^3+^ with the second process likely being Mn^3+^ to Mn^4+^. This finding is consistent with an earlier work showing Mn^2+^, Mn^3+^, or mixtures of Mn^2+^/Mn^3+^ present in the electrolyte following a high voltage hold despite the presence of Mn in the 4+ state in the cathode material itself.^13^ It also should be noted that since Mn^3+^ is EPR silent, we cannot rule out the presence of Mn^3+^ in the post voltage hold electrolyte. In fact, it may be a mixture of Mn^2+^/Mn^3+^ according to Banerjee *et al.*(14)

Further analysis of the voltammetric data, as shown in Figure 3, can yield insight into the electrochemical as well as potential solution phase reactivity of the Mn complexes formed during Mn dissolution from LMO. In Figure 3a, there is strong asymmetry in the peak current for the oxidation at 3.3 V and the reduction peaks at 3.2 and 2.7 V. The peak current for the oxidation process is significantly higher than that shown for the subsequent reduction peaks. This asymmetry potentially indicates that the products formed at 3.3 V may undergo further solution phase reactions, however, the aforementioned possibility of interactions with the underlying substrate cannot be ruled out. To explore this further as well as to determine whether further solution-based processes may be changing the properties of degradation products as they diffuse from the cathode/electrolyte interface further into the cell, we varied the tip to substrate spacing. Figure 3d compares CVs collected with the tip electrode in bulk solution (several millimeters from the LMO surface) with identical parameters to that collected at a distance of ∼20 μm from the substrate. Note that in bulk solution, a significant decrease in the oxidation peak current at 3.3 V and a loss of the curve crossing phenomenon for the process occurring at 4.5 V were observed. The asymmetry at 3.3 V was still observed at both tip to substrate distances. This clearly indicates that the origin of the asymmetry is not strictly due to an interaction with the underlying substrate but is likely tied to a solution phase reaction of the oxidized species generated at 3.3 V. The origin of the increased current near the LMO substrate is not clear at this time but could be due to a higher concentration of dissolution products at the cathode/electrolyte interface than that in bulk solution or possibly an interaction with the underlying substrate.

As mentioned, the presence of the observed asymmetry for measurements in bulk solution strongly implies that the reaction products formed at 3.3 V undergo further solution phase reactions once formed. It is possible that the oxidation products degrade before the reduction sweep leading to the decreased peak current for associated reduction processes. It may also be possible that a solution phase reaction may be regenerating Mn^3+^ and there by augmenting the oxidation current. The data presented here are unable to definitively assign cause to either potential mechanism. However, it appears to remain clear that the LMO degradation products likely undergo complex solution phase processes after leaving the cathode. We are further exploring these processes currently.

Based on previous work,^39−41^ it is expected that the lithium salt anion in an electrolyte may impact the degradation of the LMO model cathode film. To examine this, a series of voltage hold experiments was conducted in varied electrolyte formulations. The tip was positioned in bulk solution in order to avoid potential complicating interactions with the LMO film substrate. Initially, cyclic voltammetry was employed to confirm that the electrochemical behavior of the LMO model cathode was similar to that observed in Figure 1c for the two new electrolytes studied (LiPF_6_, and LiTFSI—both at 1 M concentration in PC). Figure S3 demonstrates that similar faradaic responses were observed for the LMO films for both lithium salts with no evidence of side reactions with the LMO substrate up to 4.6 V.

**Figure 4:**
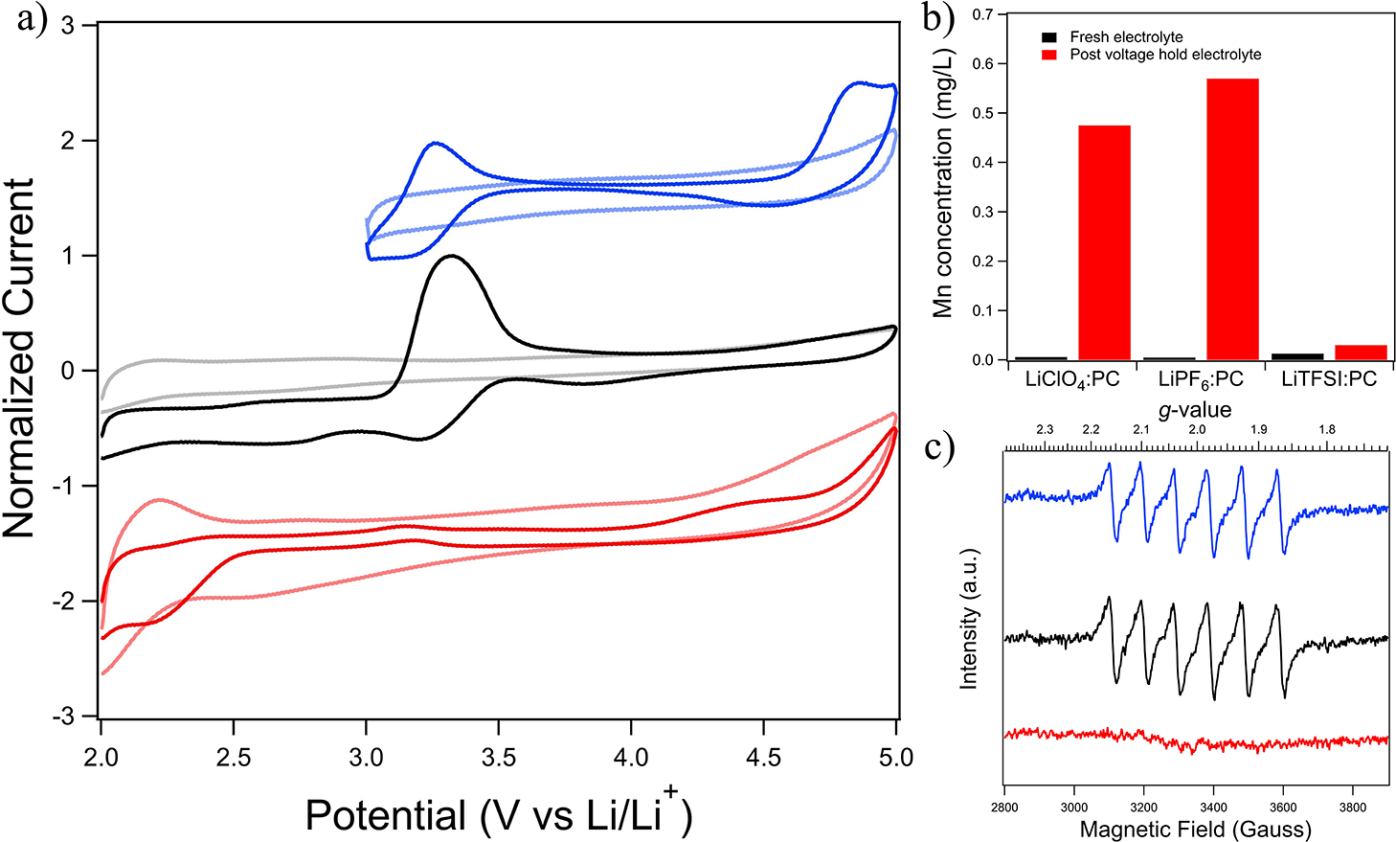
(a) Normalized SECM tip electrode voltammograms collected over an LMO substrate on SS with the tip held in bulk solution above a LMO thin film on SS in LiPF_6_/PC (blue), LiClO_4_/PC (black), and LiTFSI/PC (red) (dark color, after 4.5 V hold and light color, before voltage hold) at the scan rate of 1 V/s using a 25 μm Pt tip. Data were normalized by dividing both the initial and post voltage hold scans by the maximum current observed in the post voltage hold data for each salt sample. (b) ICP analysis of each electrolyte fresh (black) and after voltage hold (red) and (c) EPR signals for various electrolyte solutions [LiPF_6_/PC (blue), LiClO_4_/PC (black), and LiTFSI/PC (red)] after voltage hold.

Figure 4a compares the tip CVs collected in 1 M LiClO_4_, LiPF_6_, or LiTFSI in PC (LiClO_4_/PC, LiPF_6_/PC, and LiTFSI/PC, respectively) before and after 4.5 V holds of the LMO substrate. Data were collected from 2 to 5 V for the LiClO_4_ and LiTFSI electrolytes, while data for the LiPF_6_ system were only collected from 3 to 5 V. This was carried out to avoid an observed background electrolyte reaction for the LiPF_6_ electrolyte occurring below 3 V. Both CVs of LiClO_4_ and LiPF_6_ show obvious signal for electroactive degradation products following the 5 h voltage hold. Specifically, the CV for the LiPF_6_-based electrolyte displays a similar trend to the LiClO_4_ electrolyte with an oxidation peak observed at ∼3.3 V and a subsequent reduction peak at ∼3.2 V. Note, however, that the asymmetry observed in the case of the LiClO_4_ appears to be absent in the LiPF_6_-containing electrolyte. For the PF_6_^–^ anion-containing electrolyte, the peak reduction current observed at 3.2 V is almost identical to the peak oxidation current at ∼3.3 V, while the CV of LiClO_4_/PC is asymmetric, as discussed earlier. This observation appears to indicate that the Mn complex formed in the PF_6_^–^ anion-containing electrolyte is potentially more stable from an electrochemical or solution-based reactivity perspective. The CV of the LiPF_6_/PC-based sample also shows a second redox process above ∼4.5 V, which appears to show irreversible behavior. In the case of the TFSI^–^-containing electrolyte, the voltammogram collected after the 5 h voltage hold shows distinct features that do not appear as observed in the case of the PF_6_^–^- and ClO_4_^–^-containing electrolytes.

Figure 4b shows ICP data comparing the Mn concentration changes before and after the high voltage hold for each electrolyte system studied. ICP data were also collected for fresh electrolytes to establish any possible baseline Mn contamination that may be present. The Mn concentrations in all three fresh electrolytes are extremely low (0.006 mg/L, <0.005 mg/L, and 0.013 mg/L, for the LiClO_4_/PC, LiPF_6_/PC, and LiTFSI/PC electrolytes, respectively). After the high voltage hold, the concentration of Mn in LiClO_4_/PC and LiPF_6_/PC increased to 0.475 and 0.570 mg/L, respectively. The Mn concentration in LiTFSI/PC only increased to 0.030 mg/L but was measurable above earlier mentioned background concentrations, suggesting that the Mn dissolution in LiTFSI/PC does occur but may be relatively mild and kinetically sluggish compared with the other two electrolytes. EPR results of the electrolytes after the high voltage hold experiments are displayed in Figure 4c. Both LiClO_4_/PC and LiPF_6_/PC show six hyperfine lines for Mn^2+^, while no signal was observed for LiTFSI/PC. In summary, similar results for Mn dissolution were observed in LiClO_4_/PC and LiPF_6_/PC, while the TFSI^−^-based electrolyte appeared to show significantly different behavior.

We examined two hypotheses that may explain the different behavior in the LiTFSI/PC system. First, considering that the increase in Mn concentration in LiTFSI/PC following the high voltage hold is modest, it may not be detectable with our SECM and EPR protocols as they presently exist. Second, the degradation in LiTFSI/PC may undergo a different pathway, which generates disparate degradation products containing Mn^3+^ rather than Mn^2+^. EPR may show no observable signal in the LiTFSI/PC results due to the fact that Mn^3+^ is EPR silent, as discussed earlier. If we are seeing Mn^3+^ rather than Mn^2+^, we would also expect a different electrochemical response in the voltammetric measurements, as we observe.

**Figure 5:**
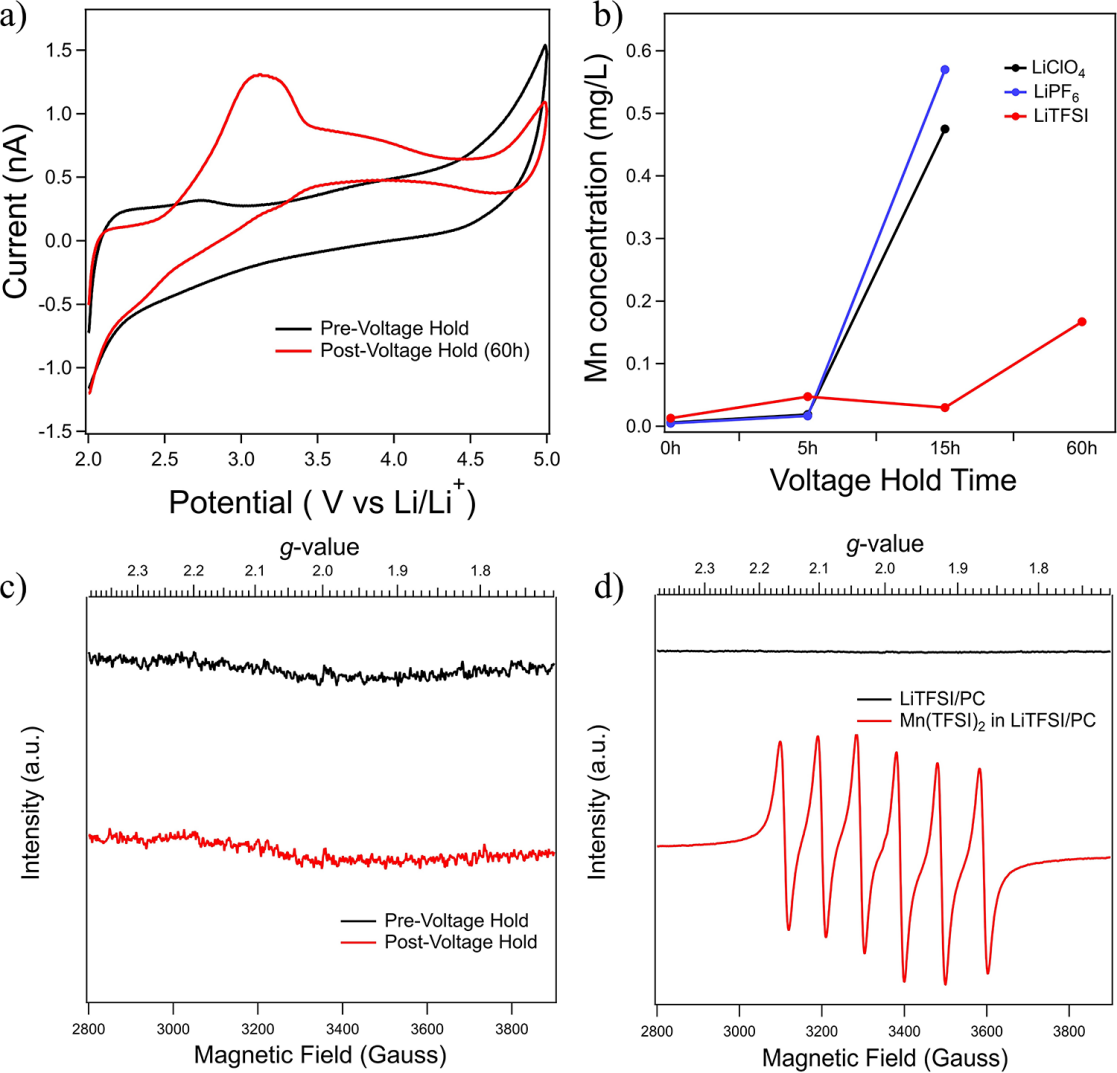
(a) SECM tip electrode voltammograms collected in bulk solution over a LMO substrate on SS in LiTFSI/PC following a 60 h, 4.5 V hold at the substrate. The tip was held several millimeters above the substrate with data collected at a scan rate of 1 V/s using a 25 μm Pt tip, (b) ICP analysis of each electrolyte over voltage hold time, (c) EPR signals for LiTFSI/PC of fresh and post voltage hold, and (d) comparison of EPR signal in the LiTFSI/PC electrolyte with and without a deliberately added Mn(TFSI)_2_ salt (Mn concentration is 94.0 mg/L based on ICP).

To explore these hypotheses further, a longer-term voltage hold experiment up to 60 h was carried out specifically for the LiTFSI/PC system. Figure 5 demonstrates the results including G/C SECM voltammograms, ICP, and EPR. After a 60 h 4.5 V hold of the LMO in LiTFSI/PC, G/C SECM tip voltammetry data now show some apparent changes in the voltammetric behavior with an oxidation process showing up at ∼3.2 V, possibly similar to what was shown in the LiPF_6_/PC and LiClO_4_/PC systems; however, the observed response still remains substantially different. Figure 5b compares the Mn concentration from ICP measurements conducted at varying durations of LMO high voltage hold for all three electrolytes. While the Mn concentrations in the LiPF_6_/PC and LiClO_4_/PC systems increase significantly after 15 h, the Mn concentration in LiTFSI/PC still remains low. After 60 h, the Mn concentration in LiTFSI/PC increases to over 0.15 mg/L but still is not as high as the other two electrolytes after 15 h. The data in Figure 5b clearly show that Mn dissolution still occurs in the TFSI^–^-containing electrolyte, however, at a significantly slower rate than that in the LiClO_4_/PC or LiPF_6_/PC systems. Note that earlier G/C SECM data for 5 h voltage hold experiments in LiClO_4_/PC and LiPF_6_/PC clearly showed significant voltammetric signal, even though Mn concentrations appear relatively low by ICP at that time. This appears to indicate that the G/C SECM method is sensitive enough to have detected Mn species that were generated in the earlier LiTFSI/PC system after high voltage hold. Based on this observation, the differences observed for the LiTFSI/PC system compared to the LiClO_4_/PC and LiPF_6_/PC systems are not strictly based on Mn concentration.

Figure 5c shows EPR analysis for the LiTFSI/PC electrolyte following the 60 h voltage hold compared with fresh electrolyte that had not been exposed to LMO. Interestingly, even in the presence of Mn (confirmed by ICP), there is no signal observed by EPR, ruling out that Mn ions in the LiTFSI-based electrolyte exist in the 2+ oxidation state. As a further control experiment, we have added a Mn(TFSI)_2_ salt directly to 1 M LiTFSI in the PC electrolyte and conducted both ICP and EPR analyses. Figure 5d compares these results to the EPR data collected from the fresh electrolyte. In the case of deliberately adding Mn^2+^ ions (by addition of Mn(TFSI)_2_ salt), an extremely prominent hyperfine structure is observed (*A* = 98 G, *g* = 2.006), suggesting that TFSI^–^ in and of itself will not hinder the Mn^2+^ measurement by EPR. The fact that ICP shows the presence of Mn after the 60 h voltage hold, but EPR shows no signal for Mn^2+^, likely confirms that the Mn-oxidation state following dissolution in 1 M LiTFSI/PC is Mn^3+^.

Given the unique phenomenon observed in the LiTFSI/PC electrolyte, we believe that the Mn dissolution process in the LiTFSI-based electrolyte may undergo a different mechanism from those in LiClO_4_- or LiPF_6_-based electrolytes. It is known that PF_6_^–^ in the presence of residual H_2_O undergoes the following reactions^42^
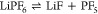
2
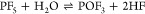
3in addition, LiClO_4_ is likely to decompose at high voltage through the following possible reactions^43,44^
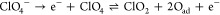
4
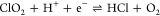
5

Both of these potential reactions generate acid within the cell which is believed to accelerate the Mn dissolution process. LiTFSI is not expected to generate acid and has been reported to have excellent stability in the presence of H_2_O, even being applied as a lithium source in aqueous electrolytes.^45−47^ The HF and HCl, decomposition products from LiPF_6_ and LiClO_4_, will likely attack the LMO and accelerate Mn dissolution, potentially driving the Mn disproportionation reaction (eq 1), which converts Mn^3+^ to Mn^2+^ and Mn^4+^. Therefore, with the potential presence of acids in the LiClO_4_/PC and LiPF_6_/PC systems, Mn dissolution is accelerated and generates the Mn^2+^ species, potentially *via* the disproportionation reaction, as we observe in the LiClO_4_/PC and LiPF_6_/PC systems. In the LiTFSI/PC electrolyte, the lack of acid appears to slow the Mn dissolution process and potentially leads to the presence of Mn^3+^ due to lack of the disproportionation reaction.

## 4 Conclusions

Efforts were made to characterize degradation products from a model thin film LMO cathode as a function of electrolyte composition, using a combination of methods including SECM, ICP, and EPR. A model LMO cathode was successfully prepared by the PAD method with confirmation by XRD and SEM. Electrochemical characterization including half-coin cell testing and cyclic voltammetry confirms that LMO on SS functioned appropriately in various electrolytes, making it a suitable model cathode system for further studies.

SECM, combined with ICP and EPR, was successfully applied to monitor the degradation of LMO in varied electrolytes. G/C SECM voltammograms were collected to characterize the electrochemical properties of LMO degradation products, while ICP and EPR were used to monitor the concentration and oxidation state of evolved Mn species. G/C SECM results show that multiple electrochemically active species are generated after holding the LMO substrate at 4.5 V for varying times in different electrolytes. Studies conducted in varied electrolyte compositions confirm that the Mn dissolution process from LMO is highly dependent on the identity of the anion present in the electrolyte. The presence of PF_6_^–^ and ClO_4_^–^ anions clearly accelerate Mn dissolution, likely through generation of acid, either through hydrolysis of the PF_6_^–^ anion to generate HF or the oxidation of the ClO_4_^–^ anion to produce HCl. Comparison to electrolytes containing only the TFSI^–^ anion still show Mn dissolution, but at a substantially slower rate due, we believe, to the lack of potential for acid generation in that system.

In addition, the identity of the anion and likely associated acid generation has a strong impact on the reactivity of Mn complexes formed following dissolution from LMO at high voltage. In the case of the PF_6_^–^ and ClO_4_^–^ containing electrolytes, we observe Mn^2+^ by EPR while in the case of TFSI^–^, it appears that we only see the Mn^3+^ species. This could potentially be explained by the presence of a disproportionation reaction in the presence of acid that converts Mn^3+^ to Mn^2+^ and potentially Mn^4+^, although our data appear to rule out Mn^4+^ being present in solution. The disproportionation reaction appears to be absent in the case of the non-acid-generating LiTFSI salt system, and hence, we appear to observe Mn^3+^. It is important to note that while EPR data clearly show the presence of Mn^2+^ in the PF_6_^–^ and ClO_4_^–^ containing electrolytes, we cannot rule out the presence of Mn^3+^. This leaves open the possibility that a solution-based disproportionation is the source of the observed Mn^2+^ rather than a fundamentally different dissolution process and is the focus of ongoing studies.

Analysis of cyclic voltammetry data for the Mn dissolution products generated in the PF_6_^–^- and ClO_4_^–^-based systems shows distinct responses, indicating that anion identity likely plays a key role in how Mn complexes are formed post dissolution. Mn complexes formed in LiClO_4_-based electrolytes appear to be less electrochemically stable than those formed in LiPF_6_-based electrolytes. The ClO_4_^–^-based degradation products appear to undergo additional solution phase reactions following oxidation. The nature of these reactions is not presently understood but is being studied further. A better understanding of these processes may help yield insight into how the complexes react elsewhere in the cell driving performance degradation.

Taken as a whole, these results provide insights into what are clearly complex chemical and electrochemical mechanisms related to the Mn dissolution process as well as subsequent solution phase reactions likely occurring near the cathode/electrolyte interface. Further work will continue to elucidate some of the mechanisms discussed here as well as explore potentially rapid processes occurring immediately at the cathode/electrolyte interface to further clarify these mechanisms.

## Supporting Information Available

CV curve of LiMn_2_O_4_ on SS in coin cell, charge/discharge curves of the LiMn_2_O_4_ thin film up to 500 cycles, rate capability cycling sequentially from 20 to 200 μA and back to 20 μA for every five cycles at each discharge rate within 3.4–4.3 V, cycling performances of the LiMn_2_O_4_ thin film between 3.0 and 4.3 V at 20 μA, CV curve of the LiMn_2_O_4_ film in LiClO_4_ and LiTFSI in PC electrolyte at a scan rate of 5 mV/s, using the SECM setup, where Pt wire is the counter electrode and Li metal is reference, and protocol of the standard feedback method to position tip (PDF)
