# Supplementary material for: Understanding Degradation at the Lithium-Ion Battery Cathode/Electrolyte Interface: Connecting Transition-Metal Dissolution Mechanisms to Electrolyte Composition
Source: ACS Appl Mater Interfaces. 2021 Mar 4;13(10):11930–9. doi: 10.1021/acsami.0c22235 (PMC10156081; doi:10.1021/acsami.0c22235)
Supplement: Supplementary file 1 — am0c22235_si_001.pdf [file am0c22235_si_001.pdf]

Supporting Information

Understanding Degradation at the Lithium-Ion Battery Cathode/Electrolyte Interface: Connecting Transition Metal Dissolution Mechanisms to Electrolyte Composition

Di Huang[1,2], Chaiwat Engtrakul[1], Sanjini Nanayakkara[1], David W. Mulder[1], Sang-Don Han[1] Meng Zhou[2] Hongmei Luo[2] and Robert C. Tenent[1,3]*

[1]National Renewable Energy Laboratory, Golden, CO 80401, United States

[2]Department of Chemical and Materials Engineering, New Mexico State University, New Mexico 88003, United States

[3]Renewable and Sustainable Energy Institute, University of Colorado, Boulder, Colorado 80303, United States

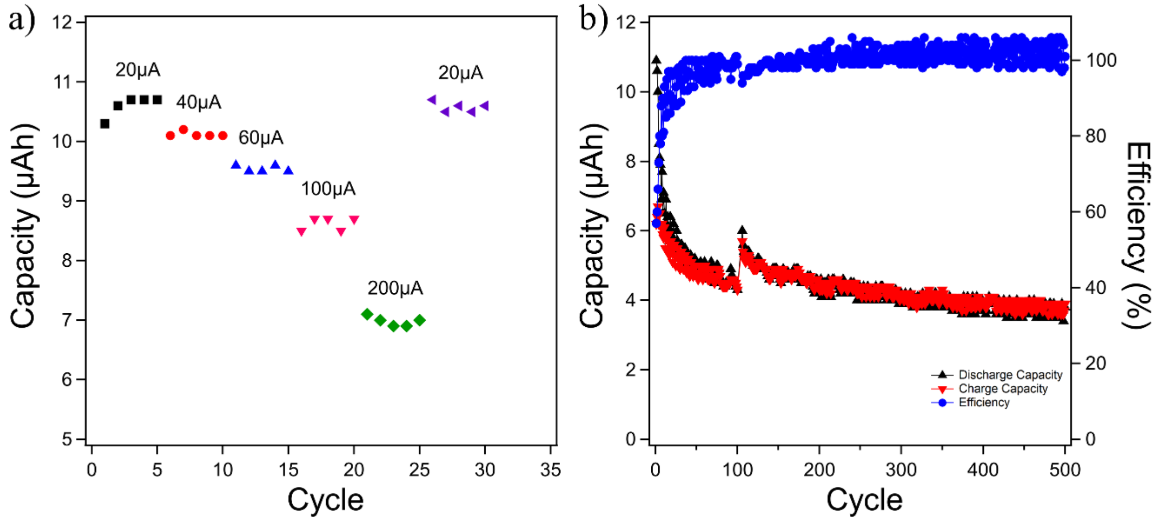

Figure S1. a) Rate capability cycling of PAD LMO model cathodes collected sequentially from 20 μA to 200 μA and back to 20 μA for every 5 cycles at each discharge rate within 3.4 - 4.3 V, b) charge/discharge curve of $LiMn_2O_4$ thin film up to 500 cycles.

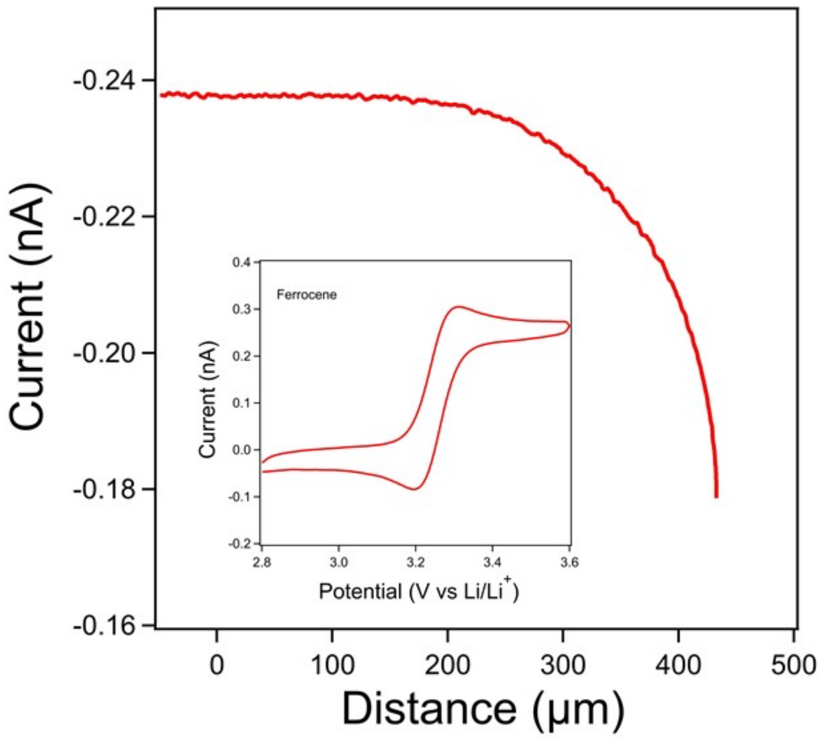

Figure S2. Approach curve of a 25 μm Pt SECM tip electrode over an unbiased LMO substrate using the ferrocene/ferrocinium redox couple. Inset figure shows CV of the ferrocene/ferrocinium redox couple collected at the tip electrode prior to substrate approach in $LiClO_4$:PC at the scan rate of 1 V/s.

SECM analysis was conducted with the tip positioned both near the active substrate surface as well as far from the substrate in "bulk" solution. SECM tip positioning was achieved using a negative feedback approach method. **Figure S2** shows an SECM current vs distance approach curve as well as cyclic voltammogram collected at the tip electrode for the ferrocene/ferrocenium redox couple in 1 M $LiClO_4$. The cyclic voltammogram shows the expected steady-state sigmoidal shaped current vs. voltage response for oxidation of ferrocene. Tip positioning was achieved by biasing the tip electrode at 3.45 V vs Li/Li^+ in order to drive the steady-state, diffusion-limited oxidation of the ferrocene redox mediator. The substrate electrode was not biased. As expected for a negative feedback response, the tip current decreases as the tip electrode approaches the underlying unbiased substrate due to physical blocking of diffusion of the ferrocene redox mediator to the tip surface. The tip was allowed to approach the substrate until the tip current decreased to 75% of that measured at "infinite" distance from the LMO substrate. Based on the standard negative feedback approach curve, this would correlate with achieving a tip to substrate spacing of ~ 20 μm for the 25 μm tip electrode. [1] After the tip approach, the cell was then flushed with fresh electrolyte 5 times and another cyclic voltammogram was collected at the tip electrode to confirm that no signal from the ferrocene/ferrocenium couple was observed prior to conducting further measurements. Positioning in "bulk solution" was conducted simply by placing the tip electrode in the cell electrolyte solution.

Figure S3 shows cyclic voltammetry data collected to confirm the functionality of the PAD LMO films in 1M $LiClO_4$ and 1M LiTFSI in PC. A similar response is seen to the previously demonstrated data shown in (**Figure 1c**). Peak definition is less clear in Figure S3 because the CVs in $LiClO_4$ and LiTFSI were conducted at a higher scan rate (5 mV/s *vs* 0.1 mV/s).

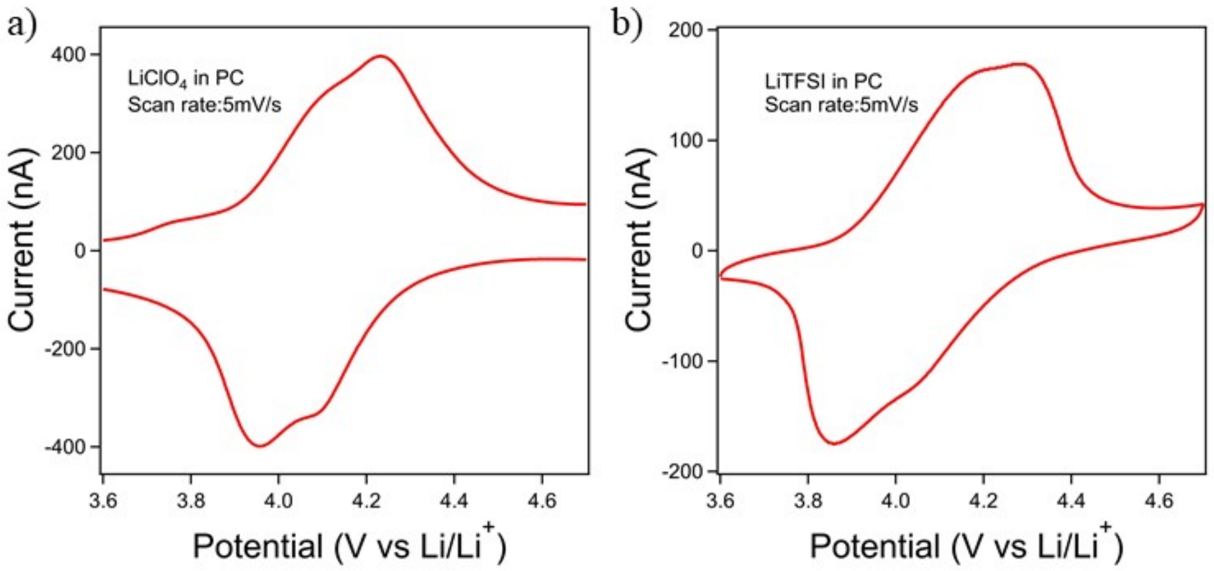

Figure S3. The CV curve of $LiMn_2O_4$ film in a) $LiClO_4$ and b) LiTFSI in PC electrolyte at the scan rate of 5 mV/s, using the SECM setup where Pt wire is counter electrode and Li metal is reference.

Reference

1. Mirkin, M. V., *Scanning Electrochemical Microscopy*. 2nd ed.; CRC press: 2012.
